# Dislocation of the Head of the Radius Forwards

**Published:** 1863-10

**Authors:** F. M. Weller

**Affiliations:** Evanston, Ill.


### DISLOCATION OF THE HEAD OF THE RADIUS FORWARDS.

By F. M. WELLER, M. D., Evanston, Ill.

On the 30th July last, Miss G., came to me for treatment for an injury to the left elbow. She had, about two hours before, while engaged in some game of amusement with several companions, slipped and fallen, but could give no definite account of the manner of striking. There was a bruise over the left scapular region, and another at the elbow joint, outside and a little below the olecranon, directly over and behind the head of the radius in its normal position. The forearm was flexed about 45 degrees from a straight line, the hand supinated.

On trial I found voluntary extension and flexion limited to an arc of 45 degrees, while forcible extension was impossible beyond 22½ degrees, and flexion 90; at these points the motion being suddenly checked as if by a lock. Voluntary pronation and supination were readily performed though to a limited extent; but the supine position was the least uncomfortable, and if the hand was left free, while the attention of the patient was diverted, was involuntarily assumed. The head of the radius could not be distinctly felt anywhere, but a slight depression, scarcely distinguishable to the eye, was perceptible to the touch when the head was sought for in place and in the middle third of the forearm the parallelism of the two bones could not be satisfactorily made out, the superior end of the radius not being as distinctly felt as in the other arm.

From these facts my diagnosis was, a dislocation of the head of the radius forwards beneath the tendons of the biceps, and lodged in a cavity of the humerus. I proceeded to the reduction without an anæsthetic, as follows: The patient was seated on a chair, her right hand grasping that of a friend; my assistant, (a gentleman wholly unacquainted with surgical operations,) standing behind the chair, and grasping the left arm so as to make counter extension as directed; I took a seat in front and at the left, with my right hand grasping the wrist. I held the hand so as to bring the bones parallel, and flexed the forearm within 22½ degrees of a right angle, then with my left hand placed beneath the fore arm at its upper third, so as to place my fingers on the posterior, and thumb on the anterior surface, I pressed the radius forward to free it from the humerus, and then separated it as far as possible from the ulna, and with my thumb pressed it outwards and backwards, at the same time extending the forearm with my right hand, while my assistant made counter extension by firmly holding the arm.

There was no click or snap during the operation, and not very much pain, but extension and flexion were at once perfectly restored. The head of the radius could be distinctly felt in its normal position. I placed the arm in a sling and directed frequent and free bathing with cold water about the injured joint; informed the young lady she must expect considerable stiffness of the joint for a long time, but cautioned her against any attempt at voluntary extension lest the bone might again be thrown out, before the wounded ligaments should have become perfectly healed. There was considerable swelling about the joint, and for thirty-six hours a good deal of pain, but the treatment was closely followed and the joint rapidly improved. At the time of writing (Sept. 1st.) all perceptible stiffness has disappeared.

I believe the mode of reduction adopted in this case differs from that usually directed in the surgical works, and I am not aware it has before been described or practiced; and that it may have advantages worthy of consideration.

